# Identifying barriers, facilitators, and implementation strategies for a faith-based physical activity program

**DOI:** 10.1186/s43058-020-00043-3

**Published:** 2020-06-08

**Authors:** Jessica Haughton, Michelle L. Takemoto, Jennifer Schneider, Steven P. Hooker, Borsika Rabin, Ross C. Brownson, Elva M. Arredondo

**Affiliations:** 1 Institute for Behavioral and Community Health, San Diego State University, 9245 Sky Park Court, Suite 221, San Diego, CA 92123 USA; 2 ServiceNow, 3260 Jay St, Santa Clara, CA USA; 3 College of Health and Human Services, San Diego State University, San Diego, CA USA; 4 Department of Family Medicine and Public Health, University of California, San Diego, USA; 5 Prevention Research Center in St. Louis, Brown School, Washington University in St. Louis, St. Louis, MO USA; 6 Department of Surgery (Division of Public Health Sciences) and Alvin J. Siteman Cancer Center, Washington University School of Medicine, Washington University in St. Louis, St. Louis, MO USA; 7 School of Public Health, San Diego State University, San Diego, CA USA

**Keywords:** Health promotion, Faith-based, Physical activity, Latinos/Hispanics, Community, Evidence-based interventions, Implementation strategies, Scale-up, Dissemination, Qualitative

## Abstract

**Background:**

Community engagement is critical to the acceleration of evidence-based interventions into community settings. Harnessing the knowledge and opinions of community leaders increases the likelihood of successful implementation, scale-up, and sustainment of evidence-based interventions. *Faith in Action* (*Fe en Acción*) is an evidence-based *promotora*-led physical activity program designed to increase moderate-to-vigorous physical activity among churchgoing Latina women.

**Methods:**

We conducted in-depth interviews using a semi-structured interview guide based on the Consolidated Framework for Implementation Research (CFIR) at various Catholic and Protestant churches with large Latino membership in San Diego County, California to explore barriers and facilitators to implementation of *Faith in Action* and identify promising implementation strategies for program scale-up and dissemination. We interviewed 22 pastors and church staff and analyzed transcripts using an iterative-deductive team approach.

**Results:**

Pastors and church staff described barriers and facilitators to implementation within three domains of CFIR: characteristics of individuals (lack of self-efficacy for and knowledge of physical activity; influence on churchgoers’ behaviors), inner setting (church culture and norms, alignment with mission and values, competing priorities, lack of resources), and outer setting (need for buy-in from senior leadership). From the interviews, we identified four promising implementation strategies for the scale-up of faith-based health promotion programs: (1) health behavior change training for pastors and staff, (2) tailored messaging, (3) developing community collaborations, and (4) gaining denominational support.

**Conclusions:**

While churches can serve as valuable partners in health promotion, specific barriers and facilitators to implementation must be recognized and understood. Addressing these barriers through targeted implementation strategies at the adopter and organizational level can facilitate improved program implementation and lead the way for scale-up and dissemination.

Contributions to the literature

- This research examines the barriers and facilitators to implementation of a faith-based *promotora*-led physical activity promotion program.
- The Consolidated Framework for Implementation Research (CFIR) was used to identify factors affecting program implementation of health promotion programs in faith-based settings.
- Findings provide specific suggestions on implementation strategies for scale-up of health promotion programs in faith-based settings.

## Introduction

Despite the well-established evidence for physical activity (PA) in the prevention and control of cancer and other chronic diseases, approximately 80% of US adults and adolescents are insufficiently active [1–4]. In particular, only 13.8% of Latinas (women of Latin American descent) meet the PA guidelines, compared to 23.7% of non-Latina white women [5]. Furthermore, Latinas engage in fewer total minutes of PA than Latino men (19 vs. 30 min/day) [6]. Dissemination and implementation of evidence-based PA programs are needed to help mitigate health disparities among Latinas and other at risk groups [7].

Faith-based organizations (FBOs) provide a unique setting to implement evidence-based interventions (EBIs) and are increasingly recognized as important partners in Latino-focused health promotion efforts, including PA [8–12]. The strong connection between health messages and the spiritual mission of the FBO, in addition to its reach into underserved communities, further justify the church as an effective health promotion setting [13, 14]. Studies have documented the effectiveness of health promotion programs in FBOs; however, significant gaps exist in translating EBIs into faith-based settings to have broader reach [15, 16]. Understanding the contextual factors is a critical step in translating research to practice, particularly in community settings [17]. Engaging stakeholders in the process of translation can lead to more effective assessments of contextual factors influencing implementation, and therefore more effective translation of behavioral interventions [18].

*Faith in Action* (*Fe en Acción*) is a faith-based multi-level *promotora-*led intervention promoting moderate-to-vigorous PA (MVPA) among Latina women through group PA classes and Motivational Interviewing (MI) calls [19, 20]. The 2-year evidence-based intervention consists of the following core components: six group PA classes offered each week (cardio dance, strength training, and walking groups) led by *promotoras* at participating churches and MI calls consisting of identifying barriers to PA and goal-setting every 4 months. *Promotoras*, also known as community health workers, lay health advisors, and peer educators, have shown to be effective in promoting health and providing health education for underserved populations in many parts of the world [21–23], particularly among Latino populations [24–27]. Given their connection to the community, *promotoras* are uniquely able to integrate health promotion activities and connect culturally and linguistically with members of the target population [28–30]. The effectiveness of *Faith in Action* was tested using a cluster randomized controlled trial in 16 churches [31]. At 12 months, there were significant increases in accelerometer-based MVPA and self-report leisure-time MVPA among Latinas in the intervention versus comparison condition, which suggests a greater increase in PA than many other PA interventions [32]. Intervention participants, compared to those in the attention-control condition, had a 66% higher odds of meeting the PA guidelines, reduced BMI, and used more behavioral strategies for engaging in PA compared to the attention-control condition participants. Attendance to PA classes was associated with increased self-report leisure-time MVPA; number of Motivational Interviewing calls was associated with meeting the PA guidelines. There were approximately 25,000 attendees in 8 intervention churches over the course of the 5-year study. Efficacy-effectiveness trials have promoted PA in FBOs, but *Faith in Action* is the only one that has focused on Latinas [33–36].

Because the outcomes paper focused on participant outcomes, little is known of how the organizational context contributed to the success (the “how” and the “why”). Understanding the organizational context is critical for scale-up and dissemination of evidence-based programs [37]. Midway through the implementation of *Faith in Action*, we collected data examining implementation outcomes of a subset of churches participating in the PA intervention [38]. The findings suggest that pastor support, innovation-values fit, and resource availability were factors that impacted implementation effectiveness (average 6-month participation rates in PA classes at each church). In addition, churches with lower parishioner engagement had low support from church staff and leaders, while churches with higher parishioner engagement reported high pastor support, high innovation-values fit, medium to high recourse availability, and medium to high parishioner engagement [38]. While these findings shed light on the organizational-level factors affecting implementation of *Faith in Action*, more targeted studies are needed to identify determinants that impact implementation and implementation strategies that target these determinants among a broader range of religious denominations for wider scale-up and dissemination.

While churches are promising venues for health promotion programs [39–42], including PA interventions [8, 11, 43–45], the lack of understanding of organizational context and determinants for implementation and sustainment limit dissemination capability for these programs. Few faith-based health promotion studies have been taken to scale and [46, 47] most have targeted African-American populations [46, 48, 49], and only a few have targeted PA as a main outcome, with limited effectiveness [8, 11]. Webb and colleagues explored faith leaders’ perceptions of health and wellness; however, their sample was entirely Caucasian and majority Methodist, and did not focus on the implementation of a particular program [50]. Finally, few faith-based studies have systematically explored the perspectives of pastors and church staff regarding barriers and facilitators to implementation. Bernhart and colleagues administered surveys to pastors of churches participating in the Faith, Activity, and Nutrition program; however, the sample was comprised of churches with predominantly African-American membership [51]. The authors acknowledged the limitations of survey data and reported that in-depth interviews would have further elucidated the findings, explaining the mechanisms behind the barriers and facilitators to implementation.

The objective of this study is to examine factors associated with the implementation and sustainment of an evidence-based PA intervention (*Faith in Action*) through pastors and staff interviews and identify implementation strategies specific to the church context to inform future program scale-up and dissemination.

## Methods

### Study design

We conducted a qualitative study to identify barriers, facilitators, and implementation strategies for an evidence-based, faith-based PA intervention in Latino churches. The study received approval from the Institutional Review Board at San Diego State University.

### Theoretical framework

The semi-structured interview guide was developed using the Consolidated Framework for Implementation Research (CFIR) [52]. This framework was chosen because it is comprehensive, examines domains influencing implementation effectiveness, and is well suited for complex, multilevel interventions [53, 54]. Open-ended questions assessing all five CFIR domains (intervention characteristics, inner setting, outer setting, process, and characteristics of individuals) were adapted from those presented in the online interview guide tool (cfirguide.org) to assess implementation and the domains from Schell and colleagues [55] to assess sustainability. While the domains and constructs included in the CFIR are more oriented towards a healthcare setting, there are few options of frameworks aimed at examining best practices for health promotion in faith-based settings [56]. The questions were adapted to fit non-clinical settings, simplifying language to make it relevant for community-based settings and incorporating appropriate Catholic or Protestant vocabulary (e.g., parishioner, churchgoer, priest, pastor). A copy of the interview guide has been included in the supplemental files (see Additional file 1).

### Sampling and data collection

To gather data on both the previous implementation of *Faith in Action* and potential for dissemination and scale-up, we recruited pastors and staff from the 8 Catholic churches that participated in the *Faith in Action* intervention and 10 Protestant churches in San Diego County serving large Latino congregations. Protestant is the second most common denomination for churchgoing Latinos [57]. These other churches were identified using online search engines (search terms “San Diego” and “Spanish church” or “*iglesia*”) and compiled into a database of Protestant churches in San Diego County with at least one Spanish-language weekend service (*n* = 53). We used phone, email and mail for initial contact and follow-up, stopping at 5 contact attempts per church. In addition, research staff visited 10 churches to solicit interviews, prioritizing bilingual churches with large congregations and monolingual Spanish churches. This process continued until we recruited 10 churches that represented a variety of denominations within the larger category of Protestant (i.e., Evangelical, Baptist, Seventh-Day Adventist, non-denominational), demographics, and contexts to ensure variability across sites. During recruitment, we described to potential participants the purpose of the research in identifying barriers, facilitators, and strategies to the implementation of health programs in churches. Of the 61 total churches approached (8 Catholic and 53 Protestant), 18 churches, represented by 22 pastors and staff, agreed to participate. Reasons for not participating included lack of response after multiple contacts and limitations on the pastors’ and church staff time to participate in an interview.

### Data collection

The majority of the interviews (*n* = 13) were conducted by JH (a female MPH/MA researcher with experience implementing faith-based interventions) and EA (a female PhD health behavior researcher). The remaining 5 were conducted by MT (a female PhD researcher with extensive qualitative experience) and JS (a female graduate student with training in qualitative methods). Several co-authors (JH, EA, MT, JS) trained in qualitative methods, conducted the interviews in pairs. Interviews were conducted in-person at the churches in either English or Spanish according to the interviewees’ preference and lasted an average of 45 min. In some cases, more than one individual participated in an interview, resulting in 18 interviews with 22 total participants. Interviews were audio-recorded, transcribed verbatim, and reviewed by the interviewer to ensure accuracy. Field notes were collected during the interview and added to the data file. Identifying information such as pastor names were removed or abbreviated and Spanish transcripts were translated to English using standard protocols [58]. To ensure all aspects of the qualitative research was reported, the consolidated criteria for reporting qualitative studies (COREQ) [59] was used as a checklist (see Additional file 2).

### Data analysis

Data were analyzed and coded using an iterative-deductive approach consisting of a systematic and repetitive process using CFIR as the guiding theory [52]. MT and JH coded the first two interviews to develop preliminary codebooks guided by CFIR constructs. Transcripts were independently coded to categorize data and then discussed in person to reach consensus [60–62]. The initial interview codebook was used by the larger research team (JH, MT, JS, and 6 students) to code each subsequent interview transcript. Groups of three team members, including at least one of the lead researchers (JH, MT, JS) coded each transcript independently and then met in person to discuss discrepancies until consensus was reached. As necessary, the team revised the codebook, adding codes that emerged throughout the process and refining definitions in the codebook for greater clarity [63]. All changes were discussed and agreed upon in weekly team meetings to further ensure mutual understanding. Furthermore, consistency was ensured by repeated rounds of re-coding the same transcript after clarifying differences. After coding all transcripts, we identified sub-codes to categorize and re-categorize codes into relevant relationships [64]. A detailed audit trail of codebook drafts, coded and re-coded transcripts, meeting notes, codebook edits, and resolved discrepancies was kept throughout the process. We coded all transcripts by hand and then inputted the coded data to Dedoose software (SocioCultural Research Consultants, LLC version 7.5.9) to organize and sort the data.

Once the data was coded and organized, JH reviewed and examined the codes, merging similar codes and separating broader ones, sorted them into themes, and selected key quotes that represented the main themes. A group discussion involving EA and MT finalized the main themes and identified the salient barriers and facilitators (see Table 1).

**Table 1:** Barriers and facilitators to implementation of a faith-based physical activity program (*n* = 22)

CFIR domain	Barriers and facilitators to implementation	Seminal quote
Characteristics of individuals	• Pastors/staff lack self-efficacy for and knowledge of PA • Pastors have influence over churchgoers’ behaviors	“They never taught us that in priest school.” “[The pastor] is a half-marathon runner, he’s being a little modest but you know they always see him in his warm up suit - people get more motivated.”
Inner setting—culture	• Churches can support a culture of overeating and unhealthy behaviors • Churches implement programs aligned with their mission and values • Church culture and norms are influenced by pastors and staff	“[In] the Hispanic culture everything is about food because you know when they sit around food they talk, fellowship, very intimate for people.” “We can’t do everything, we want to keep us focused on doing the things that we know, that we should be focused on to make our vision a reality.” “I get in trouble because I say ‘you know what some of us don’t look like temples we look like cathedrals.’”
Inner setting—implementation climate	• Churches have many competing priorities • Many churches lack sufficient space and personnel • Programs typically come from within the church	“You know, there are many programs out there, really good programs.” “The first thing the pastor wants to know is, is this going to be more work for me?” “One of the things we don’t do a lot is have somebody from the outside come in and start a ministry.”
Outer setting	• Denominational support is critical for program success	“Well if you could get it to come from the top because I will be there but a lot of [pastors who] won’t find the time unless it’s coming from the Diocese.”

After identifying the barriers and facilitators to implementation and sustainment, JH and EA reviewed the main themes and compared them to evidence found in the literature. Through numerous discussions with the co-authors, the four implementation strategies were selected. JH and EA then used the Expert Recommendations for Implementing Change (ERIC) strategies [65] to identify and link the mechanisms of action for each implementation strategy. These were then validated by BR, a co-author with expertise in implementation science (see Table 2).

**Table 2:** Proposed implementation strategies for faith-based health promotion programs

Implementation strategy— ERIC [55] strategy	Barriers to implementation	Facilitators to implementation	Mechanisms of action
**(1) Health behavior change training***—*training and education	Pastors lack self-efficacy for PA	Pastors influence churchgoers’ behaviors	Pastors’ increased self-efficacy for PA; pastors role model healthy behaviors, including PA
**(1) Health behavior change training**—education	Pastors lack knowledge in promoting PA	Pastors provide individual-level counseling to members	Pastors encourage churchgoers to be active and healthy (e.g., praise those who meet PA goals)
**(1) Health behavior change training**—motivate change	Churches can support culture of overeating and unhealthy behaviors	Pastors influence church culture and norms	Pastors implement policies that promote health (e.g., healthy tips in church bulletins); establish a health ministry
**(2) Tailored messaging**—tailor strategies	Programs typically come from within the church	Churches implement programs that are aligned with their mission	Pastors consider the program to be relevant (e.g., social justice) to them and the church
**(3) Foster community collaboration**—develop partnerships	Churches lack sufficient space and personnel for programming	Local organizations with capacity for PA programming	Stronger collaborations with local organizations (e.g., joint projects, sharing resources/staff)
**(4) Gain denominational support***—*involve executive leadership	Denominational support is needed for a program to succeed	Denominational support can lead to wider scale-up	Denominational leadership (e.g., Diocese) encourages pastors to promote PA in churches

## Results

### Description of study participants

Eighteen semi-structured interviews were conducted with 22 pastors and staff between April and August 2018 out of 61 churches invited. Differences were not observed between interviews with one participant and those with two participants. Most interviews were conducted in English (*n* = 14) and the remainder in Spanish (*n* = 4). Seventeen of the interviewees were male and 5 were female. Of the 22 participants, all held formal positions within their churches: 11 were senior pastors or priests, 5 were associate or assistant pastors, and 6 were paid church staff members (business managers, secretaries, and deacons). One interviewee (assistant pastor) declined to be recorded and was not included in the final analysis.

### Barriers and facilitators to implementing *Faith in Action*

The barriers and facilitators identified fell within three of the five CFIR domains (i.e., characteristic of individuals, inner setting, and outer setting). We summarized major findings according to CFIR domains and included seminal quotes as illustrations of each (Table 1).

#### Characteristics of individuals

##### Pastors and staff lack self-efficacy for and knowledge of PA (barrier to implementation)

In general, pastors and staff reported having low self-efficacy for PA and lacked knowledge of PA. While a few mentioned having personal PA habits (e.g., cycling, running), most reported struggling to find time to be active and unsure of how to encourage churchgoers to be active. One pastor described hesitancy in encouraging PA, “Well probably I’m not the best person to talk about that because I need it badly. I lost already 24 pounds but I need to lose another 40 pounds.” Some pastors mentioned that they would feel like “hypocrites” if preaching about health, given their own challenges in maintaining healthy habits. Pastors shared that their demanding and unpredictable work schedules contribute to unhealthy habits and a lack of priority of their own health. In addition, pastors’ lack of self-efficacy for PA limits their ability to motivate healthy behaviors among churchgoers.

##### Pastors have influence over churchgoers’ behaviors (facilitator of implementation)

Church staff felt that pastors who were role models for PA and healthy eating had a strong influence on churchgoers. Some pastors said they had opportunities to counsel churchgoers, but while they felt equipped in mental health counseling, they were sure about how to best encourage individuals to be physically active. One pastor described his influence over his congregation in the following way, “If I open my mouth, they’ll hear. They’ll listen.”

#### Inner setting

##### Churches can support a culture of overeating and unhealthy behaviors (barrier to implementation)

Pastors and church staff described the important role of food in fellowship in the church. Participants stated that food is often served at church events and lacks healthy options. One pastor told us, “There’s a little saying in our Spanish ‘Don’t trust a skinny Pastor’” when describing the gifts of food given him by churchgoers.

##### Churches implement programs aligned with their mission and values (facilitator to implementation)

While many of the pastors and staff we interviewed acknowledged the importance of health and the need for more PA opportunities for their members, many emphasized the need for any program to align with the church’s mission and values. Given that a church’s mission is to build faith and share the message of God, finding a way to frame PA within that mission is essential. One pastor said, “I think our mission is to help people to have good health physically, mentally, emotionally, and spiritually. So, that is a complement to our mission.” Pastors did see an opportunity in connecting physical health with spiritual and mental health, as one shared, “the church is sometimes too spiritual, but the people do not die due to the spiritual, instead it is physical. They get sick because of physical health.” Finally, pastors had the view that any new program, to be successful, would have to “nestle itself into the homeostasis of the church, so it just becomes natural.”

##### Church culture and norms are influenced by pastors and staff (facilitator of implementation)

A few pastors reported having spoken about physical health from the pulpit; however, most reported not having the knowledge or resources to do so. One pastor shared, “that’s what I gotta get my hands on, some of these templates that tie in the scriptures with you know healthy eating and exercise and stuff like that.”

##### Churches have many competing priorities (barrier to implementation)

While pastors and staff recognized the importance of physical health and acknowledged the potential role of the church in promoting health among churchgoers, they also expressed that churches have many other important priorities. Pastors talked about kids and youth programming, marriage counseling and retreats, spiritual formation, social justice campaigns, and many other ongoing programs. In addition, pastors in more urban and low-resource communities shared about their challenges getting churchgoers to be involved in and committed to church programming given the transitional nature of their communities.

##### Churches often lack sufficient space and personnel (barrier to implementation)

Many of churches included in this study have small programming budgets, limited space, and few paid personnel. Pastors expressed concern about adding more to already overburdened staff and volunteers, saying, “the people who kind of are the natural leaders are already pretty spread out.” Most were also concerned about the cost of program implementation and said they have little funds to spend on programming outside of faith formation activities. Others were concerned that a new program may have to compete with current programs for space.

##### Programs typically come from within the church (barrier to implementation)

Particularly among Protestant churches, but to some extent in Catholic churches as well, most programs are implemented from within the church. Pastors described processes in which new programs are suggested by church members and leaders, vetted by the leadership, and implemented if seen as beneficial to members and sufficient resources are available. While no pastors said they would not accept an outside program, many expressed hesitancy in hosting a program from an outside organization and said they would want to hear from other pastors about their experience with *Faith in Action* before moving forward.

#### Outer setting

##### Denominational support is critical for program success (facilitator for implementation)

In this study, pastors noted the importance of support from upper denominational leadership. Pastors of Catholic churches mentioned the importance of buy-in from the local diocese. Those in Protestant churches saw denominational support as an opportunity to disseminate the program more widely to other churches.

### Promising implementation strategies for faith-based health promotion programs

The following four organizational-level implementation strategies are recommended to engage pastors and church staff to improve implementation of faith-based health promotion programs. While these strategies are tailored to faith-based settings, each is based on ERIC implementation strategies [65] as noted in Table 2. We also identified the proposed mechanism of action to explain how we linked barriers and facilitators to proposed implementation strategies.

1. *Health behavior change training for pastors and staff.* Pastors described a lack of self-efficacy for PA and that they would feel like “hypocrites” if preaching about physical health and wellness when their own behaviors do not align with their encouragement. They reported not know much about PA recommendations and cited very little if any education or training in health. Pastors shared that their demanding and unpredictable work schedules contribute to unhealthy habits and a lack of priority of their own health, statements that support the findings of Proeschold-Bell and McDevitt [66]. The stress and burnout pastors and church staff experience can lead to negative health consequences [67]. However, pastors have a strong influence over churchgoers who look to pastors as role models [50, 67, 68]. Some spoke about their role as counselors, particularly with issues related to mental health, but admitted lacking confidence in counseling churchgoers on physical health. Our findings support the literature describing the strong influence pastors have over churchgoers’ behaviors [67, 68]. Finally, pastors and church staff have a strong influence over the church norms and culture, which can, at times, support a culture of overeating and unhealthy behaviors [69]. This is supported in the literature where a number of studies have found higher rates of chronic disease among faith leaders. A national study of various denominations found higher rates of physical inactivity, obesity, poor dietary habits, and chronic disease among faith leaders compared to the general US population [70]. Social interactions around food have long been an important part of church culture, and often these foods are high-fat and not nutritious [69, 71]. While churches can have cultures that are resistant to change and act as barriers to healthy behaviors, there is promising research that churches providing instrumental support for PA through church-based PA programs see increases in PA among members [45]. As important decision-makers in the church, pastors and staff have the ability to adopt programs, align them with their vision, and influence the culture and norms of the church [14]. Pastors and church staff have the potential to influence church culture and norms in ways that promote health [68]. Given these findings, we suggest that faith-based health promotion programs include training and education on health behavior for pastors and influential church staff be included in the intervention, even if the pastors and church staff are not the primary program implementers. We expect that if pastors and church staff are trained in health behavior change methods, they will have increased self-efficacy for PA, be able to role model healthy behaviors including PA, encourage churchgoers to be active and healthy, and influence the norms and culture of the church to shift to include health-promoting policies (e.g., water to replace sugar-sweetened beverages at church events, establishing a health ministry).
2. *Tailored messaging.* Interviews with pastors highlighted the importance of aligning program messaging with the FBO’s mission and values, which may vary between denominations and churches. In general, pastors in the Protestant churches perceived the program as an opportunity for outreach and evangelization while pastors in Catholic churches were interested in how the program could build community among current parishioners. Depending on the church’s denomination and mission, the program messaging could be tailored to either of these objectives and include messages from scripture reinforcing the importance of taking care of one’s health. By tailoring the messaging to each church, the program is more likely to be accepted and perceived as something integrated into the mission of the organization.
3. *Fostering community collaborations.* Only a few of the eight churches that participated in *Faith in Action* sustained the program beyond the study period and for those that did, strong pastor support and collaborations with community partners were an important factor that facilitated sustainment. In addition, for those churches with fewer resources and less space to host program activities, partnerships with community organizations, including a local recreation center, facilitated implementation. Developing partnerships with organizations whose missions align with the health promotion program, for example, promoting PA, is expected to facilitate improved implementation and program sustainment. These collaborations could help support implementation through sharing of space for PA activities, shared personnel, or join projects to promote PA in their communities.
4. *Gain denominational support.* Churches tend to be hierarchical in structure, with the majority of the decision-making left to senior leaders [72]. During interviews, pastors highlighted the need for denominational support for the program to have long-term success. For example, buy-in from bishops in the Catholic Diocese or leaders of Protestant denominational groups would validate the program. Given the hierarchical structure of FBOs, support of the program from upper leadership is essential for pastors and staff to implement health promotion programs in their churches.

## Discussion

Identifying the barriers and facilitators to intervention implementation by engaging community members in an essential component of community-based health promotion; this kind of “co-learning”, in which researchers and community partners learn from each other, can result in more effective program implementation that addresses context-specific influences and factors [18]. To our knowledge, our study is the first of its kind to examine barriers and facilitators to implementation of a faith-based PA program through interviews with pastors and church staff. Data supported the value of accounting for the inner and outer contexts when implementing PA programs in faith-based settings and they may vary by church size and denomination.

Data from the interviews suggest that targeting the health practices of church leaders would be a key strategy that would facilitate implementation and sustainment of health promotion programs in churches. In all religious denominations, pastors are role models and play a critical role in defining the characteristics of church life [73, 74]. Maton found that church groups with capable leaders reported positive group assessments and well-being, and that pastors influence attendees’ commitment and perceived social support [75]. Increasing the self-efficacy of leaders to engage in PA and providing education on the benefits of PA could empower leaders to promote PA and create wellness policies in their congregation. Further, church leaders who role model PA may indirectly impact the health practices of their parishioners (e.g., seeing pastors make time for walks). To date, the implementation strategies used to translate EBIs in faith-based settings have primarily focused on ways pastors can adopt and implement program activities and less on the health practices of the pastors themselves [67, 76]. The implementation of PA programs may have more impact and be sustained by church leaders who engage in PA within a supportive organizational climate and culture.

The results highlight the importance of tailoring messages based on church context. Each church has its own culture, norms, and values. Matching program messages with the culture of the church could lead to improved implementation and sustainment. For example, an important value of the Seventh Day Adventist denomination is healthy living (e.g., members of this denomination practice vegetarianism). Tailored messages for a Seventh Day Adventist Church could connect their value for health with PA programming, perhaps even building health into the mission statement of the church. In addition, churches vary in their style and methods of communication. Some churches communicate programming through printed newsletters and others have sophisticated social media and web platforms. Strategies used to promote the program and invite churchgoers to participate should be tailored to the preferred communication method of each church.

Strengthening the links between churches and health organizations embedded in the larger ecosystem was another proposed strategy noted by participants. Local and national organizations that promote the well-being of community members like schools, parks and recreation departments, YMCA, Catholic Charities, and health departments may provide resources that would help churches implement and sustain health promotion programs [77–79]. Most participants reported little connection with outside organizations but had a desire to build those connections and viewed them as important for successful implementation and sustainment. This strategy would involve increasing the capacity of pastors to establish partnerships with outside organizations that could support the implementation and sustainment of PA programs. In this role, church leaders can also connect churchgoers and community members to resources they may not otherwise have and increase the impact of community organizations.

Lastly, participants noted the value of having denominational support when implementing health programs. When considering the Catholic denomination, strong denominational support from the Diocese would support pastors in the implementation of health programs and potentially bring in resources. For instance, the pastors could request time during their monthly meetings at the Diocese to discuss how *Faith in Action* and other health programming is impacting their church and its members. This would demonstrate to pastors that the Diocese supports health programming in churches and would encourage more pastors to devote time and resources to establishing health ministries and PA programs.

This study contributes to other dissemination and implementation work in faith-based settings [46, 47, 80]. While the potential of health promotion programs in faith-based settings has been recognized [10, 14], there is limited evidence on specific barriers and facilitators to program implementation by church leaders and implementers in faith-based settings. Wilcox and colleagues examined the adoption, reach, and effectiveness of a faith-based healthy eating and PA intervention in African-American churches in South Carolina by surveying church members of the participating churches [46]. Another paper examined the perspectives of church leaders who participated in the same intervention [51]. Church leaders identified barriers to program implementation including resistance to change, age of churchgoers, lack of participation, lack of time, weather, lack of leadership, and limited budget [51]. Facilitators included internal support, communication, leadership, external support, health opportunities, tailoring, and champions [51]. While the sample (African-American churches) and methods (survey) vary from our study with church leaders and staff of Latino churches, the barriers and facilitators echo and support our findings. Based on their findings, Bernhart and colleagues recommend providing training and technical assistance directly to church leaders and staff, which supports one of our selected implementation strategies. As noted by Leyva and colleagues, capacity building of church leaders and staff would help support adoption and implementation of health promotion programs in faith-based settings [81].

### Limitations and strengths

While the sample size meets evidence-based guidelines [82], the generalizability of the findings could be limited by the relatively small number of interviews. In addition, the findings may not be generalizable to churches that serve other communities and denominations. Of the 53 Protestant churches approached, only 10 participated in interviews. The results may not represent the opinions of pastors and church staff who did not participate.

The majority of published studies have focused on African-American churches and relied on survey data [33, 46, 51]. Unique to our study is a focus on the perceptions of pastors and staff at Latino churches of a faith-based PA promotion program. Finally, data from multiple sources (pastors and church staff) from various denominations further ensured the credibility and dependability of the findings.

The majority of faith-based health promotion studies have been conducted in Christian faiths [8, 42, 46, 47, 67, 83]. Future studies may explore adapting the proposed implementation strategies to other faiths, as they seem to be promising approaches.

## Conclusions

This study with FBO leaders about a faith-based PA promotion program yielded important information about barriers and facilitators to implementation and helped identify implementation strategies to employ in future work that supports scale-up and sustained implementation. The findings suggest that there are a number of barriers to implementing health promotion programs in faith-based settings. Incorporating implementation strategies including training in health behavior change, tailored messaging, community collaborations, and denominational support are expected to result in improved implementation and sustained use of EBIs in FBOs.

As public health practitioners seek to partner with FBOs, addressing these key barriers and facilitators through identified implementation strategies is essential. Others working in faith-based settings can use our results to develop and design interventions that address these barriers to implementation.

## Supplementary information

**Additional file 1:.** Interview guide.

**Additional file 2:.** COREQ checklist.

## Data Availability

The data that support the findings of this study are available from the corresponding author upon reasonable request.

## Abbreviations

CFIR: Consolidated Framework for Implementation Research
COREQ: Consolidated criteria for reporting qualitative research
EBI: Evidence-based intervention
ERIC: Expert Recommendations for Implementing Change
FBO: Faith-based organization
MI: Motivational Interviewing
MVPA: Moderate-to-vigorous physical activity
PA: Physical activity
