# Supplementary material for: Identifying barriers, facilitators, and implementation strategies for a faith-based physical activity program
Source: Implement Sci Commun. 2020 Jun 8;1:51. doi: 10.1186/s43058-020-00043-3 (PMC7427873; doi:10.1186/s43058-020-00043-3)
Supplement: Supplementary file 1 — Additional file 1:. Interview guide. [file 43058_2020_43_MOESM1_ESM.docx]

**Additional file 1: Interview Guide for Church Leaders and Staff**

1. **Previous Fe Implementation –skip to section B for new churches [CFIR – Intervention Characteristics]**

*As you may remember, Faith in Action was a 2-year program promoting physical activity among your church members. We hired 2-3 “promotoras” from your church to lead physical activity classes, both here at the church and in the local community. The classes were open to anyone, however we recruited and enrolled about 27 study participants whom we followed for the 2 years of the study. Our results showed an increase in minutes of physical activity, decrease in BMI, and an increase in behavioral strategies to promote PA.*

*Based on this success, we’re hoping to implement Faith in Action in more churches and want to make the program even better. Your opinions about the program and its implementation are valuable as we seek to improve the program and expand its reach.*

1. **Before Faith in Action, were there any existing programs in your church about health and/or physical activity? If so, please tell me a little about these programs.**
2. **How was the decision made to implement Faith in Action in your church?**
   1. Who was involved in the decision?
3. **What factors influenced your decision to implement Faith in Action?**
   1. Probes: needs of churchgoers, strong demand for a program like this, fits with church values/goals, other churches had them, etc.
   2. Did you know of any other churches that had physical activity or health programs?
4. **What level of involvement did you or other church leaders have in Faith in Action?**
   1. Who did you ask if you had questions about Faith in Action or its implementation?
   2. How available were these individuals?
5. **How successful would you say the program was in your church?**
   1. Please describe how you defined success.
6. **What were some of the benefits of having Faith in Action in your church?**
   1. Probes: more individuals attending church, higher attendance at other church activities
7. **What was difficult about having the program at your church?**
   1. Probes: available resources (time, space, personnel), infrastructure (social architecture, age, size, or physical layout of the church), culture (general beliefs, values, assumptions, etc.)
   2. To what extent did other programs take priority over implementing Faith in Action?
8. **If given the opportunity, would you do the program, again? Why or why not?**
   1. What would you do differently if you were to do the program again?
9. **Church Characteristics [CFIR Inner Setting]**
10. **Tell me a little about your church:**
    1. How many members/attendees do you have?
    2. What percent of that are Latino/Hispanic?
    3. Do you offer services in Spanish? How many?
    4. What programs are offered?
11. **How would you describe the culture of your church?**
    1. What are the top values of your churchgoers?
    2. What are their concerns and needs?
    3. What are the most important programs in your church?
12. **Is there a need or desire in your church for a health or physical activity program?**
    1. Please describe this need/desire.
13. **Interaction with Others [CFIR Outer Setting]**
14. **To what extent do you interact with colleagues or priests from other churches in the area? With those higher up in the denominational leadership (i.e., Diocese)? With other leaders on a national level?**
    1. What kind of information do you exchange?
    2. Do you ever go to conferences or trainings?
15. **How does your church partner with other institutions or organizations?**
    1. Is it unusual for a church to collaborate with an academic institution? Do you know any churches that do so?
    2. How comfortable would you be partnering with an organization outside of your church on a project? What do you see would be the benefits? What about the drawbacks?
       1. Probes: YMCA, Boys and Girls Clubs, outside gyms
16. **Decision-making Processes [CFIR Process]**
17. **How are decisions made in your church?**
    1. Who is involved in the process?
18. **What factors are considered when deciding what programs to implement in your church?**
    1. Probes: strong demand, fits with values/goals, other churches, cost
19. **What type of information and evidence do you typically need when considering implementing a new program?**
    1. Probes: previous success results, participant feedback
    2. What do you rely on as sources of information for this evidence?
       1. Probes: publications, testimonials, feedback from other church leaders
20. **As part of Faith in Action, the *promotoras* worked on environmental projects within the church and surrounding communities. What types of projects would motivate the church leaders to participate?**
    1. Probe: raising funds for homeless, improving church grounds, food pantry, etc.
21. **[ASK ONLY THOSE WHO DIDN’T PARTICIPATE IN EBI] Would you consider implementing a faith-based physical activity program in your church?**
    1. Why or Why not?
       1. Probes: needs of churchgoers, demand, fit with values/goals, practices of other churches, costs/resources
    2. How well would it fit with existing programs?
    3. How high a priority would this kind of program be in comparison to others?
22. **What would make it difficult to implement Faith in Action?**
    1. Probes: scope, duration, complexity, number of steps involved, disruptiveness, etc.
23. **Would events like “Family Night” (bringing together families) from time to time help engage church leaders in Faith in Action?**
    1. What would be the benefits? Drawbacks?
24. **Church Leader’s Beliefs/Values regarding PA [CFIR Characteristics of Individuals]**
25. **How important is physical health to you?**
    1. What kinds of things do you do to stay healthy?
26. **In your opinion, what is the role of churches in promoting health?**
    1. Why should churches get involved in health promotion, if at all?
27. **How interested do you think churchgoers in your church would be in participating in Faith in Action?**
28. **Who are the key individuals that would help Faith in Action to be successful?**
    1. How would suggest engaging them? (Probes: social marketing, education, training, etc.)
    2. Are there people likely to go above and beyond to ensure the success of the program? (Probe: church secretary, etc.) In what ways? How would you encourage these individuals to help implement the program?
29. **How interested or engaged would church staff be in the program? What could you do to improve engagement?**
30. **If you were to be involved in our project, how would you be able to assist churches in your community to promote health, given your position and experience?**
31. **Would you be willing to help promote the program at church events and mass?**
    1. Would you commit to a standing meeting with the church members leading the program?
32. **Would you be available to participate in a 1-2 day training for the implementation of the program? What factors or incentives should we consider in the training?**
    1. Probe: incentives for the church, etc.

**F. Sustainability**

1. **What factors would help maintain a program like Faith in Action?**
   1. Probes: financial support, community partner, etc.
2. **What resources are available in your community to support churches in their efforts to promote health?**
3. **Who else should we speak with that may be knowledgeable about churches and the Latino community and willing to share their opinions with us?**
